# D3GRN: a data driven dynamic network construction method to infer gene regulatory networks

**DOI:** 10.1186/s12864-019-6298-5

**Published:** 2019-12-27

**Authors:** Xiang Chen, Min Li, Ruiqing Zheng, Fang-Xiang Wu, Jianxin Wang

**Affiliations:** 1 School of Computer Science and Engineering, Central South University, Changsha, China; 2 Department of Mechanical Engineering and Division of Biomedical Engineering, University of Saskatchewan, Saskatoon, SK S7N 5A9 Canada

**Keywords:** Gene regulatory network, Dynamic network construction, Regression, DREAM challenge

## Abstract

**Background:**

To infer gene regulatory networks (GRNs) from gene-expression data is still a fundamental and challenging problem in systems biology. Several existing algorithms formulate GRNs inference as a regression problem and obtain the network with an ensemble strategy. Recent studies on data driven dynamic network construction provide us a new perspective to solve the regression problem.

**Results:**

In this study, we propose a data driven dynamic network construction method to infer gene regulatory network (D3GRN), which transforms the regulatory relationship of each target gene into functional decomposition problem and solves each sub problem by using the Algorithm for Revealing Network Interactions (ARNI). To remedy the limitation of ARNI in constructing networks solely from the unit level, a bootstrapping and area based scoring method is taken to infer the final network. On DREAM4 and DREAM5 benchmark datasets, D3GRN performs competitively with the state-of-the-art algorithms in terms of AUPR.

**Conclusions:**

We have proposed a novel data driven dynamic network construction method by combining ARNI with bootstrapping and area based scoring strategy. The proposed method performs well on the benchmark datasets, contributing as a competitive method to infer gene regulatory networks in a new perspective.

## Introduction

Gene regulation plays an important role in gene transcription [1, 2], gene differentiation [3], cell fate decisions [4, 5], complex diseases [6]. To elucidate the structure of gene regulatory networks (GRNs) has been a central effort of the interdisciplinary field of systems biology. With the advent of high-throughput technologies such as microarrays and RNA sequencing, tremendous amounts of data have been generated, which makes it feasible to infer GRNs from exclusive expression data or multiple classes of data based on computational methods [7]. However, inferring the GRN only from gene expression data remains a daunting task due to the small number of available measurements and the high dimensional, noisy data.

Various methods have been proposed for GRN inference [8–10], such as correlation and information theory based methods, Boolean Networks (BNs), Bayesian networks, ordinary differential equations (ODEs), and regression based methods. These approaches can be divided into two categories with different levels of granularity. The first category predicts the presence or absence of gene interactions to give a static network describing only the topological information, correlation and information theory based methods belong to this category. Other methods belong to the second category, which predicts the rate of gene interactions describing both topological and dynamic information. ODEs and regression based methods are two kinds of most widely applied techniques in all of the classes of GRN inference methods.

In correlation and information theory based methods, other than the simple Pearson correlation [11] one of the most favored metrics is mutual information (MI) [12], which is capable of capturing complex non-linear and non-monotonic dynamics between pairs or groups of genes [13, 14]. ARACNE [15] employs Data Processing Inequality (DPI) to discard indirect interactions from a triplet of genes. Subsequently based on the same purpose, conditional mutual information (CMI) [16], local overlapped gene clusters based conditional mutual information (Loc-PCA-CMI) [17], part mutual information (PMI) [18] and partial information decomposition (PID) [19] are proposed to eliminate false positive or indirect regulatory links as much as possible.

In BNs, the alternative states of a gene are represented with discrete value 0 (inactive) and 1 (active), the regulatory interactions are described by Boolean logic [20]. Probabilistic Boolean Networks (PBNs) [21] brings in probability into standard BNs to express uncertainty in the regulatory logic. Typical variants, such as Stochastic Boolean networks (SBNs) [22], aim to improve the computational performance of PBNs. BNs’s weakness is that the models only consider genes in discrete states. Thus the detail information involved in real gene expressions can not be captured effectively.

Bayesian networks, including traditional Bayesian networks [23, 24] and dynamic Bayesian networks (DBNs) [25], model the gene regulation processes based on probability and graph theory. Bayesian networks regard regulations of genes as the dependence probabilities between random variables and learn the optimal structures from gene profiles. Bayesian networks suffer from considerable computational overheads, despite recent advances [26] hence are not applicable to large genome-wide data sets.

ODEs provide an infinitesimal description of the regulation dynamics [27], by relating the rate of change (time derivative) of a gene to its expression value. Inferelator [28], S-system model [29–31] are typical approaches in ODEs. Generally, ODEs based methods are flexible by taking advantage of large parameters space estimation. As a result, akin to Bayesian networks tremendous computation is required to fulfill the task.

Most regression based methods formalize the GRN inference problem as a feature selection problem and construct the GRN with some ensemble strategy. GENIE3 [32] is recognized as state-of-the-art on some benchmark datasets [33], which is based on feature selection with ensembles of random forests. TIGRESS [34] uses least angle regression (LARS) with stability selection combined to solve the GRN inference problem. The NIMEFI method [35] explores the potential of several ensemble methods, such as GENIE3, Ensemble Support Vector Regression (E-SVR) and Ensemble Elastic Net (E-EL) [36], and combines the predictions of these methods under a general framework. bLARS [37] can be viewed as a variant method of TIGRESS, in which regulation interactions are modeled from a predefined family of functions, and the final GRN is obtained by a modified LARS algorithm with bootstrapping.

Recently, data driven dynamic network construction especially in a physical system is a pretty attractive and interesting topic. SINDy [38] assumes that there are only a few important terms that govern the dynamics so that the equations are sparse in the space of possible functions. It then uses sparse regression to determine the fewest terms in the dynamic governing equations required to accurately represent the data. ARNI [39] is a model-independent framework for inferring direct interactions in network dynamical systems, which is relying only on their nonlinear collective dynamics. It solves nonlinear systems of differential equations via functional decomposition and expansions in basis functions.

Though bLARS, SINDy and ARNI are proposed in different areas, they are somehow similar in the basic thought. Table 1 shows the comparison of these methods from three different aspects. Formal function decomposition means whether the method has a formal description with equations of function decomposition; sparse group constraints indicates whether the method utilizes sparse group constraints with the candidate terms, while network based construction indicates if the method aims to recover a whole network structure. Both SINDy and ARNI do not intend to address the problem of uncovering the physical mechanism from network level, but solely from the unit level instead. Motivated by the fact that none of the methods covers all the three points, in this study we propose a new data driven dynamic network construction method, contributing as the first attempt including above three aspects systematically. D3GRN casts the regulatory relationship of each target gene into functional decomposition problem and solves each sub problem in the way of feature selection, by using the Algorithm for Revealing Network Interactions (ARNI). The whole network structure is recovered by the bootstrapping strategy with the area based scoring method. We compare the performance of our method D3GRN to several state-of-the-art methods in DREAM4 and DREAM5 gene reconstruction challenge, and the results show our method performs competitively in terms of AUPR.

**Table 1:** Comparison of the related methods

	bLARS	SINDy	ARNI	D3GRN
Formal function decomposition	×	✓	✓	✓
Sparse group constraints	✓	×	✓	✓
Network based construction	✓	×	×	✓

## Method

### Problem definition

GRNs can be viewed as directed acyclic graphs (DAGs) if both up-streaming or down-streaming regulatory relationships among genes are not considered and the self-regulatory mechanism is ignored. In a DAG, each node corresponds to a gene and each edge represents a regulatory relationship between genes. The same as many other ensemble methods (e.g., [32], [34, 35, 40–42]), which does not utilize the information of different experimental conditions (e.g, gene-knockouts, perturbations and even replicates), we use a general framework for GRNs inference problem only based on gene expression data. As the input gene expression data, we consider the measurements for *N* genes in *M* experimental conditions. The gene expression data *A* is thus defined as follows:

1 $$  A=[x_{1},x_{2},\ldots, x_{N}] \in \mathbb{R}^{M \times N}  $$

where *x*_*i*_ is a column vector of expression values of the *i*-th gene in all the *M* experimental conditions.

GRN inference methods predict the regulatory links between genes from gene expression data *A*. Most methods provide a ranking list of the potential regulatory links from the most to the less confidence. Different DAGs can be subsequently obtained by selecting varying threshold values on this ranking list. As it is beneficial to the end-user to explore the network at all sorts of threshold levels [40], we focus only on the ranking issue in this study. Of note, the ranking is the standard prediction format of the “Dialogue for Reverse Engineering Assessments and Methods” (DREAM) [43] challenges, wherein various GRN inference methods have been proposed. Besides, we do not consider the stability of the obtained networks from the ranking.

In order to infer a regulatory network from the expression data *A*, we compute a weight score *S*_*ij*_ for a potential edge directed from gene *i* to gene *j*, where the edge indicates that gene *i* regulates gene *j* on expression level and the weight score *S*_*ij*_ represents the strength that gene *i* regulates (including both upstream regulates and downstream regulates) gene *j*.

### Network inference with ensemble regression methods

Motivated by the success of ensemble methods based on feature selection (e.g., GENIE3 [32] and TIGRESS [34]), the GRN inference problem with *N* genes can be decomposed into *N* sub problems, where each sub problem can be viewed as a feature selection issue in machine learning [44]. More specifically, for each target gene, we wish to determine the subset of genes which directly influence it from the expression level. Let *A* is the gene expression data defined in Eq. (1), the *i*-th gene is the target gene, and we define other candidate regulators with expression values in *M* experimental conditions as:

2 $$  x^{-i} = [x_{1},\ldots,x_{i-1},x_{i+1},\ldots,x_{N}]  $$

and the feature selection problem can be defined as:

3 $$  x_{i} = F(x^{-i}) + \epsilon, \forall i \in \{1,2,\ldots,N\}  $$

where *F* is any smooth, typically nonlinear function of the expression in *x*^−*i*^ of genes that are directly connected to gene *i*, and *ε* is the noise term [32, 34]. By aggregating the *N* individual gene rankings, we can obtain a global ranking of all regulatory links in a GRN.


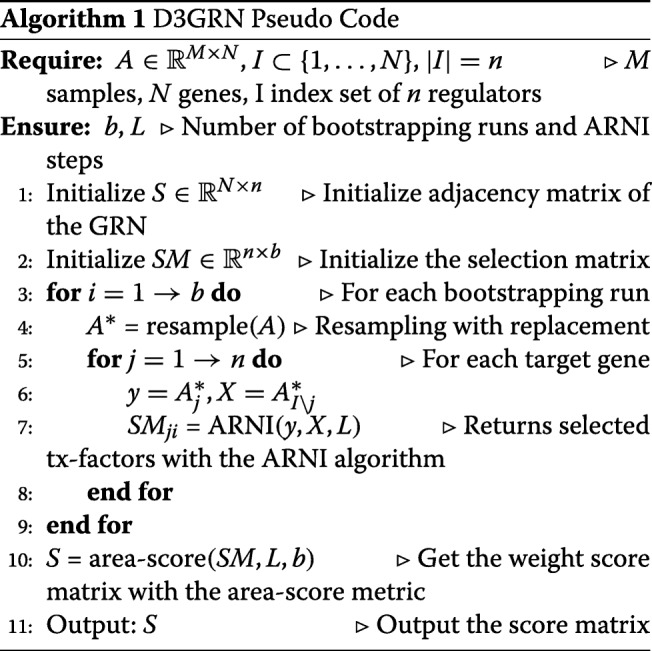


### GRN inference with D3GRN

The pseudo code of D3GRN Algorithm 1 is given below. *A*_*j*_ refers to the *j*th column of the matrix *A*, and *A*_*I*_ is the submatrix that contains only the columns in the index set *I* of *A*. Suppose that the input gene expression data matrix $A \in \mathbb {R}^{M \times N}$ and the indices of the transcription factors *I*⊂{1,…,*N*}, as well as the bootstrapping numbers and ARNI steps *L* are given. Then for each target gene *j*, and bootstrapping run *i*, by resampling the expression matrix *A* with replacement, the respective values of the target gene *j* denoted as *y*, and the expression values of the remaining transcription factors *X* are obtained, respectively. The ARNI algorithm is invoked to return an ordered list of selected transcription factors denoted by *SM*_*j*_. Finally, after all the *b* bootstrapping runs end, the matrix *SM* is passed to the area based scoring method that assigns a score between 0 and 1 to an edge between a transcription factor and a target gene. Bootstrapping and area-score techniques will be described later in this section.

#### Feature selection with ARNI

For a given unit *i* and its corresponding differential equation, ARNI turns to obtain which units *j* of the network provides direct physical interactions and appears on the right-hand side of the equation, rather than asking for details of the interaction functions among those units in the equation.

In detail, for a dynamic system with *N* units, ARNI first decomposes unit *i*’s dynamic into interaction terms with other units in the network as [39]:

4 $$  \begin{aligned} \dot{x_{i}}= & f_{i}(\Lambda^{i} x)\\ = &\sum_{j=1}^{N} \Lambda^{i}_{j} g^{i}_{j}(x_{j}) + \sum_{j=1}^{N} \sum_{s=1}^{N}\Lambda^{i}_{j}\Lambda^{i}_{s}g^{i}_{js}(x_{j},x_{s})\\ + &\sum_{j=1}^{N} \sum_{s=1}^{N} \sum_{w=1}^{N}\Lambda^{i}_{j}\Lambda^{i}_{s}\Lambda^{i}_{w}g^{i}_{jsw}(x_{j},x_{s},x_{w})+ \ldots + \epsilon_{i} \end{aligned}  $$

where $\dot {x}_{i} :=[\dot {x}_{i,1}, \dot {x}_{i,2},\ldots,\dot {x}_{i,M}]\in \mathbb {R}^{M}$, $f:\mathbb {R}^{N} \to \mathbb {R}$ is a smooth function, the diagonal matrices *Λ*^*i*^∈{0,1}^*N*×*N*^ and $\Lambda ^{i}_{j}=1$ if unit *j* directly acts on unit *i*, otherwise $\Lambda ^{i}_{j}=0$, $g^{i}_{j}:\mathbb {R} \to \mathbb {R}$, $g^{i}_{js}:\mathbb {R}^{2} \to \mathbb {R}$, $g^{i}_{jsw}:\mathbb {R}^{3} \to \mathbb {R}$ and in general $g^{i}_{j_{1}j_{2}\ldots \\j_{K}}:\mathbb {R}^{K} \to \mathbb {R}$ represents the (unknown) *K*-th order interactions between units *j*_*k*_ for all *k*∈{1,2,…,*K*} and unit *i*, the last term *ε*_*i*_ represents external noise acting on *i*.

The functions $g^{i}_{j_{1}j_{2}\ldots \\j_{K}}$ are not accessible, they can be decomposed into basis functions *h*, and we can rewrite Eq. (4) as [39]:

5 $$  \begin{aligned} \dot{x_{i}} = &\sum_{j=1}^{N} \Lambda^{i}_{j} \sum_{p=1}^{P_{1}} c^{i}_{j,p}h_{j,p}(x_{j}) \\ + & \sum_{j=1}^{N} \sum_{s=1}^{N}\Lambda^{i}_{j}\Lambda^{i}_{s} \sum_{p=1}^{P_{2}} c^{i}_{js,p}h_{js,p}(x_{j},x_{s}) \\ + &\sum_{j=1}^{N} \sum_{s=1}^{N} \sum_{w=1}^{N}\Lambda^{i}_{j}\Lambda^{i}_{s}\Lambda^{i}_{w} \sum_{p=1}^{P_{3}} c^{i}_{jsw,p}h_{jsw,p}(x_{j},x_{s},x_{w})\\ + &\ldots + \epsilon_{i} \end{aligned}  $$

where *P*_*k*_ indicates the number of basis functions employed in the expansion [45], $c^{i}_{j,p}$, $c^{i}_{js,p}$, $c^{i}_{jsw,p}$ are the unknown coefficients. Appropriate basis functions *h* are favored to form a relevant function space. For instance, the class of pairwise basis functions $g^{i}_{ij}(x_{i},x_{j})$ can be in the form of $h^{i}_{ij,p}(x_{i},x_{j}) = (x_{j}-x_{i})^{p}$ or $h^{i}_{ij,p}(x_{i},x_{j}) = x^{p_{1}}_{i} x^{p_{2}}_{j}$, etc..

Note that the framework is intended to reveal units direct interactions in dynamic systems with time series data especially. For GRN inference problem especially from non-time series data, a minor modification can be applied to Eq. (5). More specially, after replacing the left hand side time varying term $\dot {x_{i}}$ of the Eq. (5) with a non-time varying term *x*_*i*_, which is still a vector, and not accounting for self interaction meanwhile, we can have a modified equation defined as:

6 $$  \begin{aligned} x_{i} = &\sum_{j=1}^{N} \Lambda^{i}_{j} \sum_{p=1}^{P_{1}} c^{i}_{j,p}h_{j,p}(x_{j})\\ + &\sum_{j=1}^{N} \sum_{s=1}^{N} \sum_{w=1}^{N}\Lambda^{i}_{j}\Lambda^{i}_{s}\Lambda^{i}_{w} \sum_{p=1}^{P_{3}} c^{i}_{jsw,p}h_{jsw,p}(x_{j},x_{s},x_{w})\\ +&\ldots + \epsilon_{i} \end{aligned}  $$

The transformation from Eq. (5) to Eq. (6) is reasonable, and in this manner Eq. (6) is then the detail implementation of Eq. (3). The reconstruction problem then becomes identifying the non-zero interaction terms in Eq. (6). The vector of coefficients $c^{i}_{j,p},c^{i}_{js,p},c^{i}_{jsw,p}$ are unknown, hindering the retrieval of *Λ*^*i*^. It is sufficient to impose a structure of blocks of zero and non-zero coefficients in Eq. (6), representing absent and existing interactions, respectively. These structured solutions are composed by blocks $c^{i}_{s}$ of non-zero entries (representing the non-zero interactions acting on unit *i*) distributed along *c*^*i*^. The Algorithm for Revealing Network Interactions (ARNI) is proposed to solve this mathematical regression problem with grouped variables, which is a greedy approach based on the Block Orthogonal Least Squares (BOLS) algorithm [46]. ARNI can be viewed as a proper feature selection method in essence, the same as the well-known sparse group lasso [47]. Details of the algorithm are explained in the supplementary note of [39].

#### Bootstrapping

The D3GRN algorithm uses bootstrapping towards to obtain a more reliable selection of the regulators of a target gene. Bootstrapping [48] generates multiple sets of samples from the observed samples by resampling, and then computes the parameter of interest for each resampled set. Finally, an estimate of the parameter in question is obtained by averaging over all of the resampled sets. In resampling, samples are randomly selected (uniformly at random, with replacement) from the observed samples. The bootstrapping technique is frequently applied to get stable results in the case of underdetermined problems [49]. In the current D3GRN implementation, the bootstrapping runs *b* = 200 times. In each bootstrapping runs, *y* and *X* are chosen uniformly at random from resampling with replacement from the given gene expression data. Subsequently, the ARNI algorithm is utilized to select the regulators for each of these bootstrapping runs. Finally, the results of all bootstrapping runs are aggregated using an area-based scoring [34] technique. Note that the D3GRN algorithm applies bootstrapping only to obtain the high-confidence regulators for each target gene, and it does not aggregate over many bootstrapping networks such as that in [34].

#### Area-Based scoring

The area-based scoring method [37] is to assign a score to each candidate regulator with the frequency of its selection over specified bootstrapping runs. In each bootstrapping run, the ordered list of the regulators of a target gene provided by the ARNI is mathematically independent. This scoring method aims to exploit the overall ordering information about the selection of the regulators. This is achieved via the area based scoring method as follows.

Let *ϕ*_*ijl*_ be cumulative selection frequency of *j*-th regulator in the *l*-th ARNI step, *l*={1,…,*L*} and apparently *ϕ*_*ijl*_∈[0,1]. The average is taken over all bootstrapping runs, and the score *S*_*ij*_ for regulator *i* of gene *i* in total *L* steps is defined as:

7 $$  S_{ij} = \frac{1}{L} \sum_{l=1}^{L} \phi_{ijl}  $$

For example, given the values *ϕ*_*ij*1_=0.3, *ϕ*_*ij*2_=0.5, and *L*=5, the *j*-th regulator was selected 30 percent of the time in the first ARNI step and 20 percent of the time in the second ARNI step in the 5 steps. Then the cumulative selection frequency *ϕ*_*ij*2_ is 50 percent. The score *S*_*ij*_ has a natural interpretation of an area under the cumulative selection frequency curve normalized by the total area L. Clearly, this score not only takes into account the overall selection frequency of a transcription factor but also rewards the selection in the earlier ARNI steps. This method is less sensitive to the number of ARNI steps than simple ranking based on overall selection frequency *ϕ*_*ij*_.

## Results

### Input data

GRNs inference has been quite an active area of research during the past decade. Consequently, a community based consortium called “Dialogue for Reverse Engineering Assessments and Methods” (DREAM) [43] is founded. The DREAM consortium holds international reverse engineering challenges, providing standardized common input datasets and performance evaluation metrics to compare different approaches. The DREAM datasets have become a standard benchmark in the GRN inference community and are frequently used to evaluate the performance of new algorithms.

In our experiments, we use six in-silico datasets in total from both DREAM4 and DREAM5 challenges [50]. The details of the datasets are summarized in Table 2, in which columns stand for the number of genes, the number of regulators (regulatory genes), the number of samples (experiments) and the number of verified interactions respectively. If a dataset is arranged with a matrix, samples mean rows and genes mean columns. We employ five multifactorial datasets from DREAM4 challenge, with each containing 100 genes and 100 samples. The samples in these five datasets are generated from the original data by slightly perturbing all gene expression values at the same time, with the aid of the open-source GeneNetWeaver software [51]. Hence, each sample in the five datasets stands for a multifactorial perturbation experiment. Regulators can be viewed as themselves as lack of regulators provided in these small networks. We also employ one DREAM5 dataset Network 1, which is also a simulated network generated by GeneNetWeaver. The topology of the in-silico network is based on known GRNs of model organisms. Differently from that in DREAM4, The transcription factors (TFs) in DREAM5 datasets are provided as regulators which is a subset among all the genes.

**Table 2:** Detail of the datasets

Network	#Genes	#Regulators	#Samples	#Verified interactions
DREAM4 Network 1	100	100	100	176
DREAM4 Network 2	100	100	100	249
DREAM4 Network 3	100	100	100	195
DREAM4 Network 4	100	100	100	211
DREAM4 Network 5	100	100	100	193
DREAM5 Network 1	1643	195	805	4012

### Performance evaluation metrics

To evaluate the performance of the GRN inference algorithms, we use the area under the Precision-Recall curve (AUPR) as an evaluation metric. Together with AUPR, the area under the Receiver Operating Characteristic curve (AUROC) is also widely adopted for performance evaluation. In general, higher AUROC and AUPR value indicate more accurate GRN predictions. It should be noticed that, in sparse biological networks, the number of non-existing edges (negatives) outweighs the number of existing edges (positives) significantly, AUPR is more informative than AUROC [52].

We first compute the numbers of true positive (TP), true negative (TN), false positive (FP) and false negative (FN) edges by comparing the regulatory edges in the gold standard network with the top *q* edges from the ranked list output of D3GRN. The Precision-Recall curve is constructed by plotting the precision $\frac {\text {TP}}{\text {TP + FP}}$ versus the recall $\frac {\text {TP}}{\text {TP + FN}}$ for increasing *q*, *q*=1,2,…,*N*×(*N*−1), where *N* is the number of genes. AUPR is then obtained by calculating the area under the curve.

### Performance of D3GRN

The type of basis function, order *K* and the number of basis functions *P*_*k*_ in Eq. (5) play a critical role in the model decomposition in ARNI. For a large class of dynamic systems, the polynomial nonlinearities are sufficient [53]. As a reference, for gene regulatory network reconstruction in our study, the polynomial basis functions are also employed in the form of $h_{j,p}(x_{j}) = x_{j}^{p} $, and the number of basis functions is denoted as:

8 $$ P_{k}=\left\{\begin{array}{l} 5, k=1\\ 0, k>1 \end{array}\right.  $$

which means implicitly that we do not consider 2-th and above order interactions for a target gene. In fact, bLARS [37] only considers one order interaction. We also follow this way of simplification in this study. In other words, gene regulation of other genes to a target gene is a mixture of basis polynomial nonlinearities functions.

**Fig. 1:**
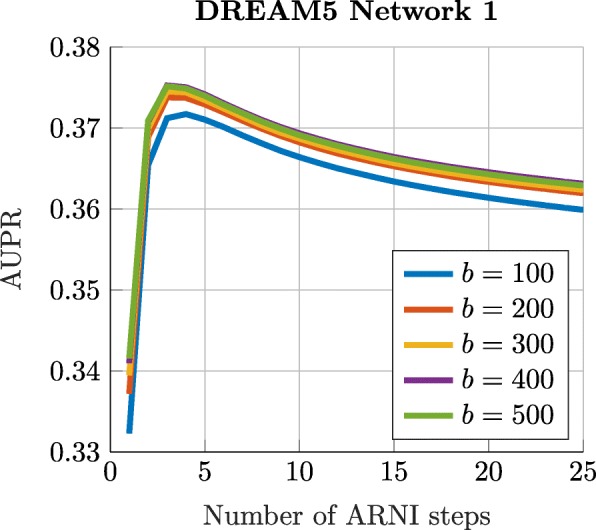
AUPR by varying ARNI steps *L* and the bootstrapping number *b* in DREAM5 Network 1

There are two parameters in D3GRN, including the number of bootstrapping runs *b* and the number of ARNI steps *L*. Figure 1 shows the effect of these two parameters by varying the number of ARNI steps and the number of bootstrapping runs from the DREAM5 Network 1. Generally, a larger number of bootstrapping runs *b* can improve the score with a sacrifice of running time. However, the performance of D3GRN is quite robust to the number of bootstrapping runs provided it is larger than a certain threshold, typically 200 runs. For the ARNI steps *L*, one’s intuition indicates that the performance would be optimal if *L* is close to the true average number of regulators in the network, which can be obtained with $\frac {2 \times \#\text {Verified interactions}}{\#\text {Genes}}$.

We have conducted two comparison experiments on DREAM4 and DREAM5 networks, to evaluate our proposed method D3GRN. NIMEFI was implemented with R, while GENIE3, TIGRESS, and PLSNET were implemented in Matlab. The codes are downloaded from the URLs provided in the corresponding papers, and we use the default values of the parameters in each method for performance comparison. Our proposed method D3GRN is also implemented in Matlab, which is available at https://github.com/chenxofhit/D3GRN.

Table 3 lists the results of D3GRN compared with other GRN inference methods on the five DREAM4 networks. In the table, the performance of D3GRN is determined with the bootstrapping number *b*=200, the number of ARNI steps *L*=2. D3GRN achieves the best AUPR value except on DREAM4 Network 2.

**Table 3:** Performance comparisons of different GRN inference methods on the DREAM4 networks in terms of AUPR

Method	Network 1	Network 2	Network 3	Network 4	Network 5
GENIE3	0.161	0.154	0.234	0.211	0.200
TIGRESS	0.158	0.161	0.233	0.225	0.233
NIMEFI	0.157	0.157	0.248	0.225	0.241
PLSNET	0.118	0.290	0.202	0.228	0.206
D3GRN	0.175	0.136	0.253	0.255	0.247

Table 4 summarizes the results of D3GRN compared with other GRN inference methods on the DREAM5 dataset. The result of D3GRN is obtained with parameters setting as the bootstrapping number *b*=200, the number of ARNI steps *L*=5 for Network 1. D3GRN achieves the highest AUPR value on Network 1.

**Table 4:** Performance comparisons of different GRN inference methods on the DREAM5 Network 1 in terms of AUPR

Network	GENIE3	TIGRESS	NIMEFI	PLSNET	D3GRN
Network 1	0.291	0.302	0.298	0.270	0.373

## Discussion and conclusion

It is reasonable to assume that interaction structure is sparse in GRN inference. Specially, under the case of “small *n* large *p*”, i.e. the small number of available samples and the large number of genes, sparsity constraints are widely considered in machine learning. In GRN, the sparsity assumption means that every gene has only a small number of regulators, which seems quite reasonable. The proposed D3GRN method also follows the same assumption. We evaluate our method on the DREAM4 and DREAM5 datasets. We hold the view that gene regulation of other genes to a target gene is a mixture of basis polynomial nonlinearities functions, which is also confirmed by the performance of our method in some extent. Theoretical or experimental analysis of this adoption is left for future work.

Another important issue is about the computational complexity of D3GRN. Speaking objectively, ARNI is suitable for small physical dynamic network recovery from the unit level. The Moore-Penrose pseudo-inverse operation of the BOLS algorithm adopted by ARNI is time consuming for large biological networks. The bootstrapping strategy in D3GRN makes it worse when dealing with large scale GRNs inference. Concerning the improvement space of ARNI, “for” loops in the bootstrapping strategy in D3GRN are completely parallelizable and can be carried out simultaneously on multiple cores and even on distributed machines in a cluster. It also deserves a try with other methods such as BOMP [46] to replace the BOLS algorithm, which is also left for future work.

The variability of the performance of the current state-of-the-art algorithms indicates that there is no algorithm that performs equally well on all datasets. However, all of these algorithms can be applied to provide inputs to a meta-algorithm that takes advantage of “the wisdom of crowds” to create a consensus and reliable community network [54, 55]. Also, the decreasing performance of all the algorithms from small networks to large networks perhaps reflects the increasing complexity of the underlying regulatory networks with varying scales. Our method advances the current state of the art, but there is still a long way to go before the issue could be treated as completely solved.

Constructing GRNs from gene expression data is an important task that can potentially contribute to our understanding of the basic mechanism such as diseases and cancers in system biology. Recent data driven dynamic networks construction methods have opened new possibilities for us to infer GRNs. In this study, we propose a data driven dynamic network construction method to infer gene regulatory networks, which transforms the regulatory relationship of each target gene into a functional decomposition problem and solves it by using the Algorithm for Revealing Network Interactions (ARNI). However, traditional data driven dynamic network recovery methods such as SINDy and ARNI do not have the ability of constructing a network. To address this limitation, we use bootstrapping and area based scoring strategy to obtain a final GRN. On DREAM4 and DREAM5 benchmark datasets, D3GRN performs competitively in terms of AUPR.

## Data Availability

A Matlab implementation of D3GRN is available at https://github.com/chenxofhit/D3GRN. The datasets of DREAM4 are available on http://www.synapse.org/#\protectSynapse:syn3049712/files/, and the datasets of DREAM5 are available on http://www.synapse.org/#\protectSynapse:syn2787209/files/.

## Abbreviations

ARNI: The Algorithm for Revealing Network Interactions
AUPR: The area under the Precision-Recall curve
D3GRN: A data driven dynamic network construction method to infer gene regulatory network
GRNs: Gene regulatory networks
